# Effect of garlic supplement in the management of type 2 diabetes mellitus (T2DM): a meta-analysis of randomized controlled trials

**DOI:** 10.1080/16546628.2017.1377571

**Published:** 2017-09-27

**Authors:** Juan Wang, Xiuming Zhang, Haili Lan, Weijia Wang

**Affiliations:** ^a^ Department of Laboratory Medicine, Zhongshan People’s Hospital, The Affiliated Hospital of Sun Yat-Sen University, Guangdong, China

**Keywords:** Garlic, allicin, *allium sativum*, T2DM, blood glucose, blood liquids, supplementary therapy

## Abstract

The present study was designed to systematically evaluate the clinical efficacy and safety of garlic supplement in the management of type 2 diabetes mellitus (T2DM). PubMed, EMBASE, the Cochrane Library, and China National Knowledge Internet (CNKI) were searched for relevant randomized controlled trials (RCTs) by using the terms garlic and T2DM up to April 2017. The quality of included RCTs was assessed by the Cochrane tool of risk of bias, and data of outcomes were pooled by REVMAN 5.3. Clinical factors were handled by meta-regression and subgroup analysis, and risk of publication bias was explored by inverted funnel plots. Nine RCTs involving 768 T2DM patients were included in the meta-analysis, and the dose of daily garlic (allicin) supplement ranged from 0.05g to 1.5g. A significant reduction in the level of fasting blood glucose in 1–2 weeks [SMD = −1.61, 95%CI (−2.89, −0.32)], 3–4 weeks [SMD = −2.87, 95%CI (−4.74, −1.00)], 12 weeks [SMD = −9.57, 95%CI (−12.39, −6.75)], and 24 weeks [SMD = −21.02, 95%CI (−32.47, −9.57)] was achieved in favour of the garlic group rather than the control group. Significantly decreased fructosamine and glycated hemoglobin (both in 12 and 24 weeks) were also found in garlic group. Meanwhile, significantly improved blood liquids of total cholesterol [SMD = −1.93, 95%CI (−2.98, −0.87), 3–4 weeks], high density lipoprotein [SMD = −0.41, 95%CI (−0.83, −0.00), 3–4 weeks] and low density lipoprotein [SMD = −3.47, 95%CI (−5.76, −1.18), 12 weeks] were confirmed after garlic administration. There was no significant difference in complications. Current data confirms that garlic supplement plays positive and sustained roles in blood glucose, total cholesterol, and high/low density lipoprotein regulation in the management of T2DM.

**Abbreviations**: T2DM = type 2 diabetes mellitus; RCT = randomized controlled trial; SMD = standard mean difference; CI = confidence interval; FBG = fasting blood glucose; HbA1c = glycated hemoglobin; HDL = high density lipoprotein; LDL = low density lipoprotein.

## Introduction

According to the analysis results of global data, the prevalence of diabetes melllitus in adults was about 153 million in 1980 and 347 million in 2008 [1]. It is estimated to increase to 439 million in 2030, with a 69% increase in developing countries and 20% in developed countries [2]. Among those cases, type 2 diabetes mellitus (T2DM) typically occurred in overweight young adults as well as aged populations [3], with the underlying mechanism a relative deficiency of insulin due to abnormal insulin sensitivity and insulin resistance involving various transport pathways and key molecules. As a common chronic disease, T2DM easily induces age-matched disabilities and multiple organ dysfunctions or failure [4]. Meanwhile, the problem of T2DM-induced depression is also proposed and focused on in recent years. It was reported that one in four patients suffered clinically significant depression, and that the depression could even adversely affect the course and increase the risk of T2DM-related complications [3]. In most standard guidelines for T2DM medical care, comprehensive treatment including diet control, exercise, drug intake, insulin injection, and sometimes bariatric surgery were recommended, with confirmed efficacy [5]. However, taking hypoglycemic drugs would be still a major and popular choice due to its flexibility and low cost for a long time, especially in developing countries where more and more newly diagnosed T2DM may emerge.

Garlic is one of the oldest cultivated plants all over the world, and is regarded as food as well as traditionally a medicine. Garlic extract is a compound of various biological activities, and proved to be beneficial for human bodies due to its antimicrobial, antioxidant, anticarcinogenic, antimutagenic, antiasthmatic, immunomodulatory, and prebiotic effects [6,7]. Previous studies have already demonstrated that it can reduce the level of blood pressure and cardiovascular events in severe hypertensive patients [8,9]. Also, it might play positive roles in the primary prevention of colorectal cancer and cardiovascular mortality, although the effects were not completely confirmed [8,10].

Currently, garlic extract is becoming one of the most extensively studied drugs, and the positive effects of garlic supplements on blood glucose control and liquid regulation were further reported, which attracted more and more attention from researchers. A series of randomized controlled trials (RCTs) of high quality were designed to investigate its efficacy in the management of T2DM during last the decades [11–20]. As a promising traditional food and medicine, together with its potential advantages of multiple targets, wide distribution, low cost, and rare complications, garlic would have a very important and significant influence on current clinical management of T2DM if its efficacy were confirmed. However, due to the limited sample size and verified outcomes, there is not yet a comprehensive and quantitative analysis with high reliability. Therefore, a meta-analysis through identifying all available RCTs was conducted to evaluate systematically the efficacy and safety of garlic supplements in the management of T2DM on blood glucose, as well as blood liquids including total cholesterol, triglyceride, high density lipoprotein (HDL), and low density lipoprotein (LDL) regulation.

## Methods

### Data sources and research strategy

The meta-analysis was prepared and reported mainly based on the preferred reporting items for systematic reviews and meta-analysis (PRISMA) guidelines [21]. Online searches were conducted in PubMed, EMBASE, the Cochrane Library, and China National Knowledge Internet (CNKI). The deadline was 15 April 2017. After reading the results of primary searches and relevant reviews, a literature search strategy was discussed and improved by all the reviewers, and final search terms included (allicin OR allicine OR allitride OR garlicin OR garlic OR allium sativum OR organosulfur OR allixin OR alliin) AND (pathoglycemia OR dysglycemia OR metabol* OR diabe* OR glucose OR sugar). Species were limited to human studies, and the languages were limited to English and Chinese.

### Study inclusion and exclusion criteria

Potential studies were included by two steps. Firstly, after removing duplicates, case series, reviews, and irrelevant studies were excluded by screening the titles and abstracts. Secondly, full texts of the remaining publications were read and assessed for four items: study design; participants; interventions; and outcome measures. Detailed criteria were as follows: (1) only studies designed as RCTs were considered, and non-randomized prospective or retrospective controlled studies were excluded; (2) participants were T2DM patients who were newly or previously diagnosed by certain methods, aged between 18 and 75 years without sex limitation, and did not have a history of serious cardiovascular disease. Patients diagnosed as hyperglycemic or hyperlipemic were excluded; (3) interventions must have been given under a random grouping situation. Comparable baselines of hypoglycemic drugs and liquid-lowering drugs needed to be enabled in each trial. Garlic was orally supplemented in the treatment group (garlic group), with a placebo or non-placebo in the control group (control group). For newly diagnosed T2DM patients garlic was administered alone, while for previously diagnosed T2DM a combination of garlic and hypoglycemic drugs or insulin was administered. Studies adopting row garlic rather than garlic extract preparations were excluded since accurate quantitative doses of garlic in each group is hard to determine; (4) the outcome measures should have at least included fasting blood glucose (FBG), glycated hemoglobin (HbA1c), other indexes of blood glucose and blood liquids, or safety (garlic related complications).

### Data extraction and outcome measurements

Information on basic characteristics in each trial was collected, which included first author, publication year, sample size, sex composition, baseline FBG level, daily dose of garlic, and outcome measured time. After extracting all available data from the included RCTs, primary outcome measurements reported by our study included FBG, plasma fructosamine, and HbA1c. Secondary outcome measurements included other blood glucose related indexes (postprandial blood glucose, HOMA-insulin resistance index, C-peptide), blood liquids indexes [total cholesterol, triglyceride, HDL, LDL], and safety.

### Methodological quality assessment

The methodological quality was assessed by using the Cochrane tool of risk of bias for RCTs [22]. To assess potential bias existing in selection, performance, detection, attrition, and reporting processes, six items were focused on: random sequence generation; allocation concealment; blinding (of participant and personal, of outcome assessment); incomplete outcome data; selective reporting; and other biases [22]. Each item was judged as low risk, unclear risk, or high risk.

During the process of study inclusion, data extraction, and quality assessment, two independent reviewers finished the work and checked the results. They resolved all disagreements by discussion.

### Data synthesis

Review Manager software (RevMan 5.3, the Cochrane Collaboration, Denmark) was used to combine the data. Because the duration of garlic administration has obvious effects on outcome measurements, subgroup analyses were performed to handle the clinical heterogeneity accordingly. After that, chi-square and *I^2^* statistical tests were used to explore statistical heterogeneity across the trials. If *I* ^2^ < 50%, statistical heterogeneity was regarded as not existing, and a fixed-effects model was chosen; otherwise, statistical heterogeneity was considered, and a random-effects model was chosen. Follow-up data of outcome measures were firstly adjusted by their baseline data, and then the value of changes was compared between garlic group and control group. To present the value of effect size, standard mean difference (SMD) was used for continuous data, and risk ratios (RR) for dichotomous data, and 95% confidence intervals (CI) and *P* value were both supplemented. *P *< 0.05 was considered to be statistically significant. To preliminarily explore the relationship between garlic administration time/dose and FBG reduction, single-factor regression analysis based on individual trials was performed by using Meta-Analyst software (for Windows 8, The Agency for Healthcare Research and Quality, USA). As for both outcome measures, different combined methods always led to different results; therefore, individual studies showing data outliers was checked, and then excluded in sensitivity analysis though changing the combined model at the same time. The trend of results was compared to show the stability and reliability of the outcomes. Risk of publication bias was explored by visually judging the symmetry of inverted funnel plots.

## Results

### Study characteristics and quality

**Figure 1.**
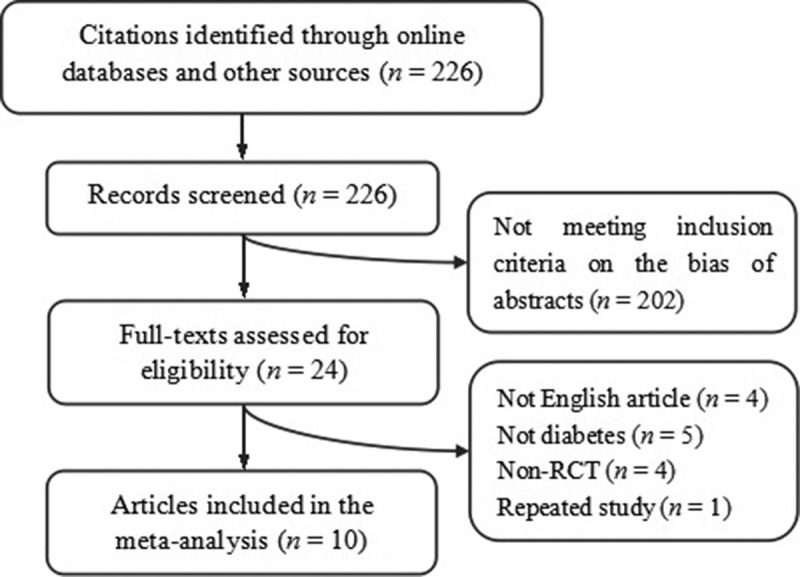
Trial inclusion.

Primary search yielded 226 abstracts. Two hundred and two of them were excluded by screening abstracts, and 14 of them were excluded by reading full texts. Finally, 10 articles reporting 9 RCTs were included (Figure 1). The combined study included 430 and 338 T2DM patients in the garlic group and the control group, respectively. As shown in Table 1, one trial had three arms [12], and they were regarded as two separate trials. The average baseline level of FBG ranged from 6.2 mmol/L to 12.2 mmol/L. Five trials adopted monotherapy of garlic for newly diagnosed T2DM, while four trials adopted a combined therapy of garlic with oral hypoglycemic drugs or insulin for previously diagnosed T2DM [12,14,16,19,20]. Daily doses of garlic ranged from 0.05g to 1.5g. Administered time ranged from 2 to 24 weeks, and outcome measured time ranged from 1 to 24 weeks for FBG, 1–4 weeks for plasma fructosamine, and 12–24 weeks for HbA1c.

**Table 1.** Baseline characteristics of the included trials.

Study	Case (n)	Sex (M/F)		Age (y)	Intervention	Baseline FBG (mmol/L)	Dose (g/d)	Outcome measured time (weeks)
		G	C					
Ashraf 2005 [11]	35/35	15/20	17/18	60 ± 5.04/58 ± 5.80	Garlic/placebo	NR	0.6	Ranged at 6, 12
Sobenin 2008 [12]	10/10	8/12		48.2 ± 2.6	Garlic/placebo	7.7 ± 0.6/7.6 ± 0.6	0.6	Ranged at 1, 2, 3, 4
	20/20	18/22			Garlic+SF/placebo+SF	9.7 ± 0.4/10.1 ± 0.8		
Sukandar 2010 [13]	32/32	5/27		54.5 ± 8.4	Garlic/control	9.7 ± 2.8	1.2, 1.6, 2.4	Ranged at 2, 4, 6, 8, 10, 12
Ashraf 2011 [14]	30/30	17/13	16/14	40 ± 5.04/35 ± 4.58	Garlic+MT/placebo+MT	7.1 ± 0.3/6.2 ± 0.5	0.9	Ranged at 12, 24
Ashraf M 2011 [15]	150/30	90/60	15/15	40 ± 5.04/45 ± 4.58	Garlic/placebo	7.1 ± 0.3/7.1 ± 0.2	0.3, 0.6, 0.9, 1.2, 1.5	Ranged at 12, 24
Song 2006 [16]	46/50	55/41		52 ± 9	Garlic+insulin/insulin	10.7 ± 2.7/12.2 ± 3.1	0.06	Post-treatment 2
Chhatwal 2012 [17,18]	30/30	NR		53.8 ± 12.2/52.8 ± 11.0	Garlic+MT/MT	8.4 ± 0.8/9.0 ± 0.9	0.5	Ranged at 2, 4, 6, 8, 12
Manafikhi 2015 [19]	51/45	NR		30–70	Garlic+MT+GB/MT+GB	12.0 ± 0.6/9.6 ± 0.5	0.05	Post-treatment 12
Atkin 2016 [20]	26/26	19/7		18–70	Garlic/placebo	-	1.2	Post-treatment 4

G, garlic group; C, control group; NR, not reported; FBG, fasting blood glucose; SF, sulfonylurea; MT, metformin; GB, glyburide.

Data were listed as mean±deviation or range.

**Figure 2.**
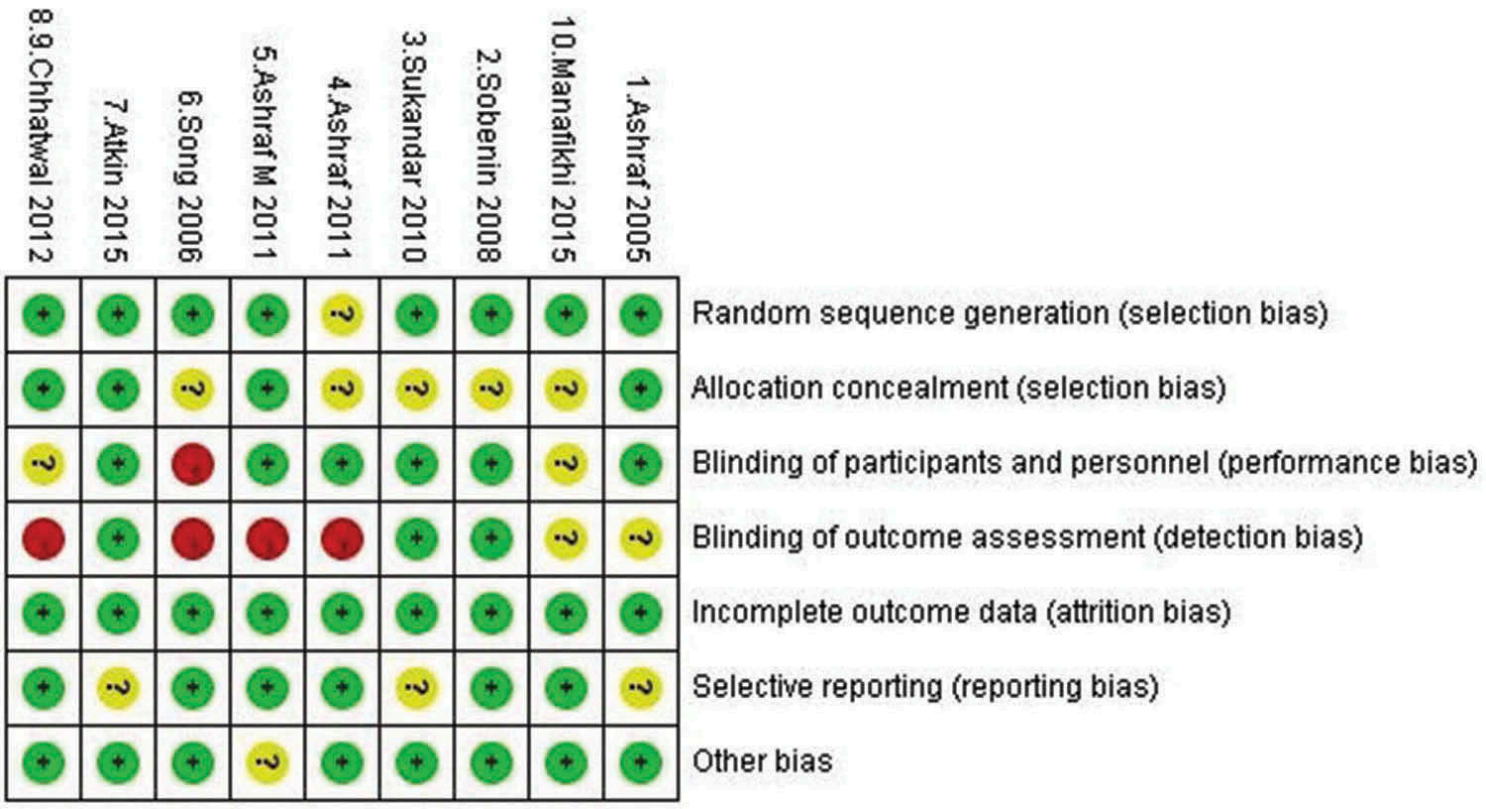
Quality assessment of the included trials.

Methodological quality assessment results showed that overall quality of the RCTs is relatively good, with moderate to low risk of bias. Five trials had unclear risk in allocation concealment [12–14,16,20], and four trials had high risk in blinding of outcome assessment [14–16,18], as shown in Figure 2.

### Effect of garlic on FBG

The data of FBG were reported in seven trials. According to the duration of garlic administration, the patients were divided into five subgroups as short-term (≦8 weeks) and medium-term (> 8 weeks) periods. For short-term effects, meta-analysis results in 1–2 weeks [SMD = −1.61, 95%CI (−2.89, −0.32), *P *= 0.01, *I^2^ *= 95%], 3–4 weeks [SMD = −2.87, 95%CI (−4.74, −1.0), *P *= 0.003, *I^2^ *= 95%], and 6–8 weeks [SMD = −0.88, 95%CI (−1.25, −0.50), *P *< 0.00001, *I^2^ *= 0%] showed that garlic led a significant reduction in the level of FBP compared with controls in random-effects model. For medium-term effects, meta-analysis in 12 weeks [SMD = −9.57, 95%CI (−12.39, −6.75), *P *< 0.00001, *I^2^ *= 98%] and 24 weeks [SMD = −21.02, 95%CI (−32.47, −9.57), *P *= 0.0003, *I^2^ *= 99%] showed that garlic achieved a sustained significant reduction in the level of FBP compared with control in the random-effects model, as summarized in Table 2.

**Table 2.** Summary of garlic on blood glucose in different combined models.

	n (G/C), *I^2^*	RE model		FE model	
		SMD (95%CI)	P	SMD (95%CI)	P
Fasting blood glucose					
1 to 2 weeks	136/140, 95%	−1.61 (−2.89, −0.32)	0.01	−0.64 (−0.91, −0.37)	< 0.00001
3 to 4 weeks	90/90, 95%	−2.87 (−4.74, −1.0)	0.003	−1.68 (−2.08, −1.29)	< 0.00001
6 to 8 weeks	60/60, 0%	−0.88 (−1.25, −0.50)	< 0.00001	−0.88 (−1.25, −0.50)	< 0.00001
12 weeks	261/255, 98%	−9.57 (−12.39, −6.75)	< 0.00001	−2.06 (−2.36, −1.76)	< 0.00001
24 weeks	180/180, 99%	−21.02 (−32.47, −9.57)	0.0003	−3.69 (−4.27, −3.11)	< 0.00001
Serum fructosamine					
1 to 2 weeks	60/60, 75%	−1.92 (−2.85, −0.99)	< 0.0001	−1.70 (−2.14, −1.26)	< 0.00001
3 to 4 weeks	86/86, 96%	−3.48 (−6.25, −0.71)	0.01	−1.47 (−1.90, −1.03)	< 0.00001
Glycated hemoglobin					
12 weeks	180/180, 98%	−6.93 (−10.71, −3.14)	0.0003	−2.75 (−3.15, −2.35)	< 0.00001
24 weeks	150/150, 82%	−13.25 (−15.83, −10.68)	< 0.00001	−12.41 (−13.48, −11.33)	< 0.00001

G, garlic group; C, control group; RE, random-effects model; FE, fixed-effects model; SMD, standard mean difference; CI, confidence interval.

### Effect of garlic on plasma fructosamine

The data of plasma fructosamine was reported in two trials. Meta-analysis results both in 1–2 weeks [SMD = −1.92, 95%CI (−2.85, −0.99), *P *< 0.0001, *I^2^ *= 75%] and 3–4 weeks [SMD = −3.48, 95%CI (−6.25, −0.71), *P *= 0.01, *I^2^ *= 96%] showed a significant reduction in the level of plasma fructosamine in the garlic group compared with control in the random-effects model, as shown in Table 2.

### Effect of garlic on HbA1c

The data of HbA1c was reported in two trials. Meta-analysis results both in 12 weeks [SMD = −6.93, 95%CI (−10.71, −3.14), *P *= 0.0003, *I^2^ *= 98%] and 24 weeks [SMD = −13.25, 95%CI (−15.83, −10.68), *P *< 0.00001, *I^2^ *= 82%] showed that garlic induced a significant reduction of HbA1c compared with control in the random-effects model, as shown in Table 2.

### Effect of garlic on other relevant indexes

Kumar et al. reported the data of postprandial blood glucose, and found that garlic group had a significant reduction compared with baseline from 2 weeks to 12 weeks [19]. Song et al. reported that C-peptide in garlic group was significantly higher than that in control group after two weeks of supplements [16]. Atkin et al. calculated the HOMA-insulin resistance index in four weeks; however, it showed a non-statistically significant difference with a boundary P value (*P *= 0.05) [17].

### Effect of garlic on blood liquids

According to the liquid composition and outcome measured time, the level of four kinds of liquids including total cholesterol, triglyceride, HDL, and LDL were compared by subgroup analyses in 1–2 weeks, 3–4 weeks, and 12 weeks. Meta-analysis results showed that garlic achieved significant reductions in 3–4 weeks in the level of triglyceride [SMD = −1.48, 95%CI(−1.89, −1.07), *P *< 0.00001, *I^2^ *= 0%] and in 12 weeks in the level of total cholesterol [SMD = −1.93, 95%CI(−2.98, −0.87), *P *= 0.0003, *I^2^ *= 34%] and LDL [SMD = −3.47, 95%CI(−5.76, −1.18), *P *= 0.003, *I^2^ *= 98%]. A significant increase was also found in 12 weeks in the level of HDL [SMD = 0.69, 95%CI (0.39, 1.00), *P *< 0.00001, *I^2^ *= 12%], as shown in Table 3.

**Table 3.** Summary of garlic on blood liquid in different combined models.

	1 to 2 weeks	3 to 4 weeks	12 weeks
Total cholesterol			
n (G/C), *I^2^*	76/80, 82%	86/86, 80%	151/145, 93%
RE: SMD (95%CI), *P*	−0.37 (−1.25, 0.50), 0.4	−0.34 (−1.06, 0.38), 0.36	−1.93 (−2.98, −0.87), 0.0003
FE: SMD (95%CI), *P*	−0.23 (−0.56, 0.09), 0.15	−0.37 (−0.68, −0.06), 0.02	−1.62 (−1.89, −1.34), <0.00001
Triglyceride			
n (G/C), *I^2^*	60/60, 80%	60/60, 0%	151/145, 91%
RE: SMD (95%CI), *P*	−0.55 (−1.41, 0.31), 0.21	−1.48 (−1.89, −1.07), <0.00001	−0.12 (−0.89, 0.64), 0.75
FE: SMD (95%CI), *P*	−0.45 (−0.83, −0.08), 0.02	−1.48 (−1.89, −1.07), <0.00001	−0.17 (−0.40, 0.07), 0.16
High density lipoprotein			
n (G/C), *I^2^*	60/60, 52%	86/86, 42%	100/100, 12%
RE: SMD (95%CI), *P*	0.47 (−0.08, 1.02), 0.09	−0.41 (−0.83, −0.00), 0.05	0.69 (0.39, 1.00), <0.00001
FE: SMD (95%CI), *P*	0.48 (0.11, 0.85), 0.01	−0.39 (−0.70, −0.09), 0.01	0.69 (0.40, 0.98), <0.00001
Low density lipoprotein			
n (G/C), *I^2^*	60/60, 95%	60/60, 87%	151/145, 98%
RE: SMD (95%CI), *P*	−0.33 (−2.27, 1.61), 0.74	0.43 (−0.69, 1.54), 0.45	−3.47 (−5.76, −1.18), 0.003
FE: SMD (95%CI), *P*	−0.74 (−1.17, −0.30), 0.0009	0.06 (−0.32, 0.44), 0.76	−2.23 (−2.56, −1.89), <0.00001

G, garlic group; C, control group; RE, random-effects model; FE, fixed-effects model; SMD, standard mean difference; CI, confidence interval.

### Safety

Three trials reported five cases of heartburn [11,15,16], and one trial reported two cases of indigestion in the garlic group and one case in the control group [18]. Meta-analysis results showed that there was no significant difference [*I^2^ *= 0%, RR = 1.96, 95%CI (0.54, 7.11), *P *= 0.31].

### Meta-regression

**Figure 3.**
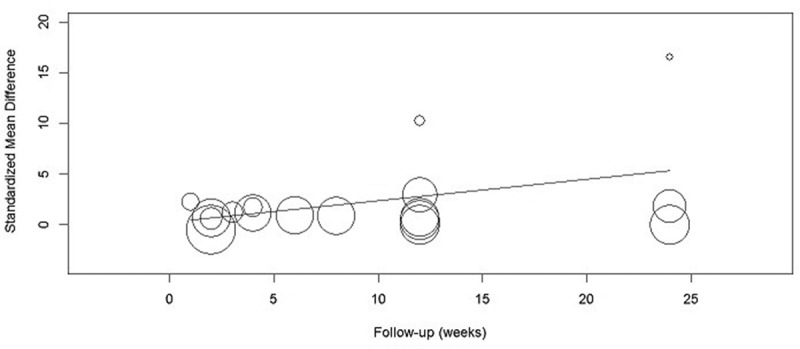
Meta-regression of fasting blood glucose and garlic administration duration.

Meta-regression in the random-effects model based on individual trials was used to preliminarily explore the possible relationship between FBG reduction and duration/dose of garlic administration. However, no significant associations with both administered duration [coefficient = 0.21, 95%CI (−0.006, 0.43), *P *= 0.056, Figure 3] and dose [coefficient = 4.22, 95%CI (−3.10, 11.54), *P *= 0.26] were found, although there was a potential trend of garlic administered duration and FBG reduction presented.

### Sensitivity analysis

All the outcomes were also analyzed by changing the combined model, including fasting blood glucose, serum fructosamine, glycated hemoglobin, total cholesterol, triglyceride, high density lipoprotein, and low density lipoprotein. Although specific data were not completely the same, the trends of most outcomes did not alter. However, total cholesterol in 3–4 weeks, triglyceride in 1–2 weeks, and low density lipoprotein in 1–2 weeks showed significant difference, indicating that unstable results may exist, as shown in Tables 2 and 3.

### Publication bias

Inverted funnel plot results showed that low risk of publication bias may exist in outcomes of blood liquids for total cholesterol, triglyceride, HDL, and LDL. For blood glucose outcomes, moderate risk of publication bias may exist in HbA1c.

## Discussion

As is reported, the compound composition in garlic is different from each other when it is prepared in different ways. Current garlic preparations mainly include aqueous homogenate of garlic, garlic power (though dehydration <60 degrees), aged garlic exact (though stored in 15% to 20% ethanol >20 months), and garlic oil (extract though steam distillation) [23]. The primary compound in garlic bulb is alliin and allinase enzyme. Allinase enzyme can be activated during the chopping or crushing process, and then it will convert alliin to allicin [23,24]. Garlic power contains only alliin, allinase enzyme, and allicin, while raw garlic and aqueous homogenate of garlic also contains adenosine [23]. Aged garlic extract contains significant new, stable, and increased antioxidant properties, such as allixin, selenium, and sallylcysteine. Garlic oil contains more active components such as diallyl, allyl methyl, and dimethyl mono to hexa sulfides [24,25].

The combined analysis included nine RCTs, in which seven adopted garlic power tablet, and two adopted aqueous homogenate of garlic [16,20], so the major compound of garlic extract was allicin. To our knowledge, this was the first meta-analysis to quantitatively evaluate the effect of garlic (allicin) for T2DM patients. And the results demonstrated that garlic significantly improved blood glucose control, and also had significantly positive roles in blood liquid regulation in 12 weeks, which is a very common co-morbility in T2DM patients.

To eliminate the influence of different baseline levels, the value of FBG reduction from baseline to outcome measured time was compared between garlic group and control group. Subgroup analysis according to different administration duration revealed that both short-term and medium-term administered garlic showed promising hyperglycemia effects from post-treatment 1 week to sustained 24 weeks. At the same time, fructosamine reflecting the average level of blood glucose in past 2–3 weeks and HbA1c reflecting the level of past 2–3 months were both significantly decreased. Among the analysed individual trials, except for two trials in 1–2 weeks [12,16], one trial in 3–4 weeks [12], and one trial in 12 weeks [14] reporting negative results of FBG, and one trial in 3–4 weeks and another trial in 12 weeks reporting negative results of fructosamine and HbA1c [17,20], respectively, all the others reported nearly the same results, although the statistical value of hypoglycemic effect differed from each other. Focused on the usage of garlic, only one trial adopted a monotherapy of garlic compared with placebo [12], while all the other trials adopted a combination therapy compared with control (such as other hyperglycemia drugs or insulin). So it was indicated that current clinical evidence mainly supported that garlic supplement would further enhance the therapeutic effect of other hyperglycemia drugs and insulin, but was not completely the significant effect of garlic alone.

The anti-diabetes effect of garlic reported in other studies would be concluded as rapid and sustained mechanisms, and among the form of garlic allicin (allyl 2-propenethiosulfinate or diallyl thiosulfinate) was considered to be the major bioactive compounds found in type-1 diabetic animal studies [23]. The rapid effect of garlic was mainly because of increased insulin secretion as well as release from pancreatic beta cells [26]. In the included studies, Song et al. reported that C-peptide in garlic group was significantly higher than baseline and control after two weeks [16]. C-peptide and insulin are both secreted by beta-cell, and they come from a same precursor: proinsulin. It was consistent with the result of previous animal studies [23], and the results to some extent indicated that immediate reduction after garlic intake and in 1–2 weeks after garlic administration were closely related to the rapid mechanism, whereas no other included trials in current meta-analysis reported the level of endogenous insulin. The sustained effect of garlic may be contributed by decreased insulin resistance. Previous animal studies reported that garlic intake more than eight weeks long relieved insulin resistance index in fructose-fed rats [27]. The included trial of Atkin et al. also focused the issue; however, the difference in insulin resistance index between the groups showed a threshold level in four weeks (*P* = 0.05) [17]. As the study only included 26 patients in each group, and measured resistance index in a relatively short duration, it was easy to explain the non-positive results. Based on the proposed rapid and sustained mechanism, further meta-regression was performed to investigate the relationship between garlic dose and duration with FBG reduction. If a rapid mechanism existed, FBG reduction may be not positively correlated with the dose of administered garlic due to insufficient pancreas function and insulin resistance. But, if a sustained mechanism existed, FBG reduction would highly positively correlate with the course of garlic administration, because insulin resistance may be gradually relieved after medium- and long-term administration. Meta-regression results showed that no statistically significant relationship was found in garlic dose and FBG reduction, as well as in garlic administration duration and FBG reduction; however, it was interesting that a clear trend was primarily presented that standard mean reduction of FBG in 1–2 weeks, 3–4 weeks, 12 weeks, and 24 weeks were 1.61, 2.87, 9.57, and 21.02. Further studies focusing on changes in the insulin resistance index during garlic administration would be very important in the future.

Besides being regarded as a metabolic disease, hyperlipidemia and hypertension were common in a large proportion of T2DM patients. They were demonstrated to severely increase the risk of macrovascular and cardiovascular events in diabetes population [5,28]. Such patients always need to take additional hypolipidemic drugs and antihypertensive drugs at the same time, and in this situation the treatment significantly increased not only the patients’ financial cost, but would also have negative influences on therapeutic compliance. Our study showed that garlic also had positive effects on improving total cholesterol, LDL, and HDL in 12 weeks. However, a previous meta-analysis reported negative results and of course proposed the high possibility of follow-up <12 weeks [29]. In the current study, all the individual trials reported positive results of total cholesterol in 12 weeks, and only one of three trials of HDL and one of four trials reported negative results in 12 weeks [17]. The difference in onset time of garlic on blood liquids may be caused by the different measured time and limited sample size in the study, thus enough courses of garlic administration may be crucial for blood liquid regulation. Low level of HDL is an independent risk factor for coronary artery disease, and increased level of HDL is protective against atherosclerotic disease [30]. Together with its confirmed anti-hypertensive effects, garlic was supposed to lower the risk of macrovascular and cardiovascular events in specific T2DM patients [9]. Meanwhile, garlic had an antioxidant effect due to a rich proportion of sulfur-containing compounds [23]; it can be also very beneficial to prevent diabetes associated complications [27].

For safety, as a traditional food as well as medicine, garlic induced very low incidence of complications (1.63%), which included mild heartburn and indigestion.

The present study has several limitations. Together with meta-regression results, we partly revealed the relationship between duration and the outcome changes, while the certain relationship between dose and outcomes was still unclear as the potential influence of the existing risk of publication bias. Although two of the included studies compared kinds of doses of garlic extract, the results should only be compared internally and not be applied to the others due to a lack of a quantitative method of bioactive compound detection. The dose of garlic to achieve hypoglycemic, hypolipidemic, and anti-hypertensive effects seemed to be different, since different rapid and sustained mechanisms may exist. Therefore, studies in the future need to explore a stable and repeatable method to define the optimal dose of garlic for T2DM patients with pure hyperglycemia or complex metabolism disturbance. Furthermore, the treatment among the trials included combined therapy and monotherapy, which was not completely consistent. And after conducting subgroup analyses, this difference together with the influence of dose would be the major sources of statistical heterogeneity. As the interventions between the groups were comparable, they would not alter the results, as only one monotherapy trial was included. Thus, the current study mostly showed that garlic as a natural plant drug had promising benefits in the supplementary or combined therapy of T2DM.

## Conclusion

The present study confirms that additional garlic contributes to improved blood glucose control in 1–2 weeks as well as in 24 weeks in T2DM, and plays positive roles in total cholesterol and high/low density lipoprotein regulation in 12 weeks. The potential sustained effects involving insulin resistance relief seems promising, however further studies are warranted to support the finding.
